# Antibody design using LSTM based deep generative model from phage display library for affinity maturation

**DOI:** 10.1038/s41598-021-85274-7

**Published:** 2021-03-12

**Authors:** Koichiro Saka, Taro Kakuzaki, Shoichi Metsugi, Daiki Kashiwagi, Kenji Yoshida, Manabu Wada, Hiroyuki Tsunoda, Reiji Teramoto

**Affiliations:** 1 Research Division, Chugai Pharmaceutical Co., Ltd, Kamakura, Kanagawa Japan; 2 Research Division, Chugai Pharmaceutical Co., Ltd, Gotemba, Shizuoka Japan

**Keywords:** Biotechnology, Computational biology and bioinformatics

## Abstract

Molecular evolution is an important step in the development of therapeutic antibodies. However, the current method of affinity maturation is overly costly and labor-intensive because of the repetitive mutation experiments needed to adequately explore sequence space. Here, we employed a long short term memory network (LSTM)—a widely used deep generative model—based sequence generation and prioritization procedure to efficiently discover antibody sequences with higher affinity. We applied our method to the affinity maturation of antibodies against kynurenine, which is a metabolite related to the niacin synthesis pathway. Kynurenine binding sequences were enriched through phage display panning using a kynurenine-binding oriented human synthetic Fab library. We defined binding antibodies using a sequence repertoire from the NGS data to train the LSTM model. We confirmed that likelihood of generated sequences from a trained LSTM correlated well with binding affinity. The affinity of generated sequences are over 1800-fold higher than that of the parental clone. Moreover, compared to frequency based screening using the same dataset, our machine learning approach generated sequences with greater affinity.

## Introduction

Antibodies are powerful tools for therapeutic and biological research in the present era^1,2^. In vitro display technology (e.g., phage display, ribosome display) is an efficient method of antibody discovery. The display system features powerful high-throughput and excellent adaptability to low immunogenic or highly toxic antigens. However, antibodies from display libraries tend to have moderate binding activity^3^. One reason for this is the limited library size^4^. Therefore, an additional affinity maturation step is necessary to thoroughly explore the sequence space.

However, affinity maturation experiments can be costly and laborious. Traditionally, clonal antibody screening is achieved by randomly picking phage clones and applying Sanger sequencing analysis. Recently, next-generation sequencing (NGS) technologies have been adapted for the in-depth evaluation of the complementarity determining region (CDR) sequence landscape^5^. However, frequently read sequences do not necessarily have high affinity. It could take time to optimize panning conditions for improving the accuracy. Therefore, there is a clear demand to find measures other than frequency when utilizing NGS derived sequences to discover promising candidates.

To address these issues, we employed a long short term memory network (LSTM)-based sequence generation and prioritization procedure to efficiently discover sequences with higher affinity. LSTM is a widely used deep generative model in natural language processing^6,7^. We used a trained LSTM model to sample virtual sequences and avoid combinatorial explosion in the sequence space. Then, we prioritized the most promising sequences according to their likelihood as calculated by the trained LSTM.

To demonstrate the effectiveness of our method, we applied it to the affinity maturation of antibodies against a hapten. In recent years, anti-hapten antibodies are expected to find use not only as research tools, but also as industrial reagents, and in diagnosis and therapy^8–10^. However, obtaining antibodies against haptens is difficult because of the limitation of antigenic epitope. Therefore, the development of more powerful screening methods for obtaining high-affinity antibodies is desired.

We confirmed that the likelihood of a sequence using a trained LSTM correlated well with binding affinity in generated sequences and demonstrated that our machine learning approach generated sequences with greater affinity compared to frequency based screening using the same dataset.

## Results

### Overall workflow

**Figure 1:**
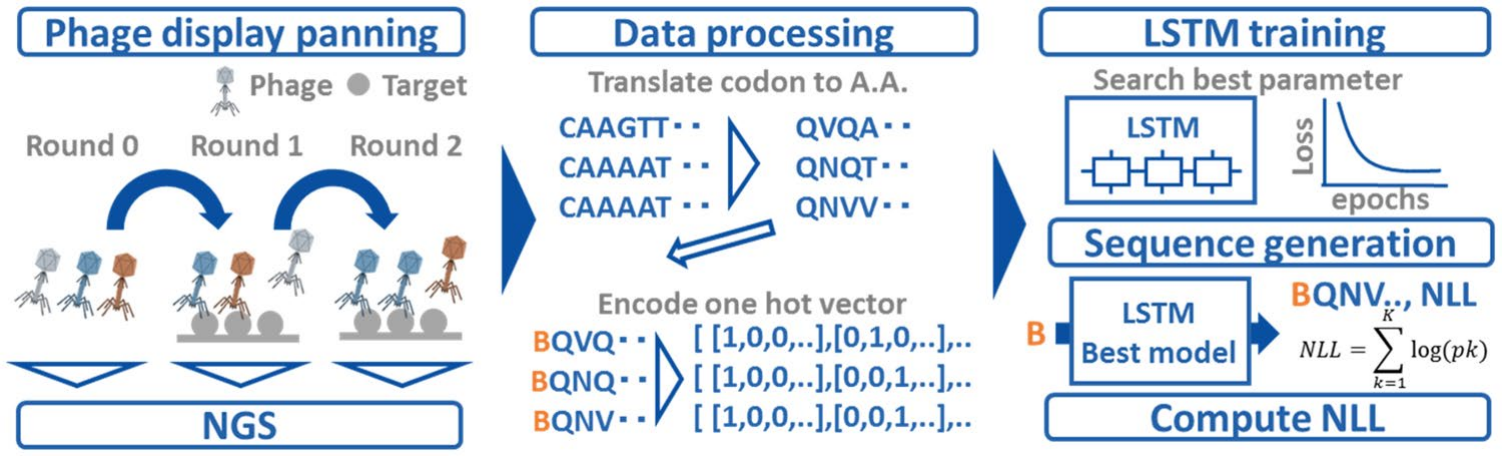
Overall workflow for our proposed LSTM based sequence generation and prioritization scheme.

Figure 1 shows the overall workflow of our LSTM based sequence generation and prioritization scheme. First, we perform panning against an antigen, e.g., kynurenine (Phage display panning), and then we get a large-scale enriched antibody sequence by NGS (NGS). Next, we translate codons to amino acids and extract VH sequences. Subsequently, we add the start token, labeled “B,” and encode the amino acids to one hot vector (Data processing). Then, we train a LSTM model from enriched sequences (LSTM training). After that, we begin generating virtual sequences that mimic enriched sequences from B based on the LSTM model (Sequence generation). Finally, we compute the negative logarithm of their likelihood (NLL) to prioritize virtual sequences (Compute NLL). After finishing the above process, we select promising sequences according to NLL.

### Training data acquisition

To validate the affinity maturation method, we applied it to an anti-kynurenine antibody. Kynurenine is a metabolite found in the niacin synthesis pathway. Indoleamine-pyrrole 2, 3-dioxygenase (IDO) catalyze tryptophan to kynurenine. It was demonstrated that the enzyme was overexpressed in many types of cancer^11^. Therefore, kynurenine accumulation is a potential biomarker for cancer and antibodies against the metabolite could be useful for cancer research and diagnosis. F02 is an anti-kynurenine antibody we previously derived from a human naïve phage display library. We constructed this F02 heavy chain-based antibody library to find potential residues for kynurenine binding. The library design is based on crystal structure analysis of the antibody-antigen complex and anti-kynurenine binding profiles of F02 mutational variants to conserve the paratope and diversify other potential residues (data unpublished, library design: Table 1).

**Table 1:**
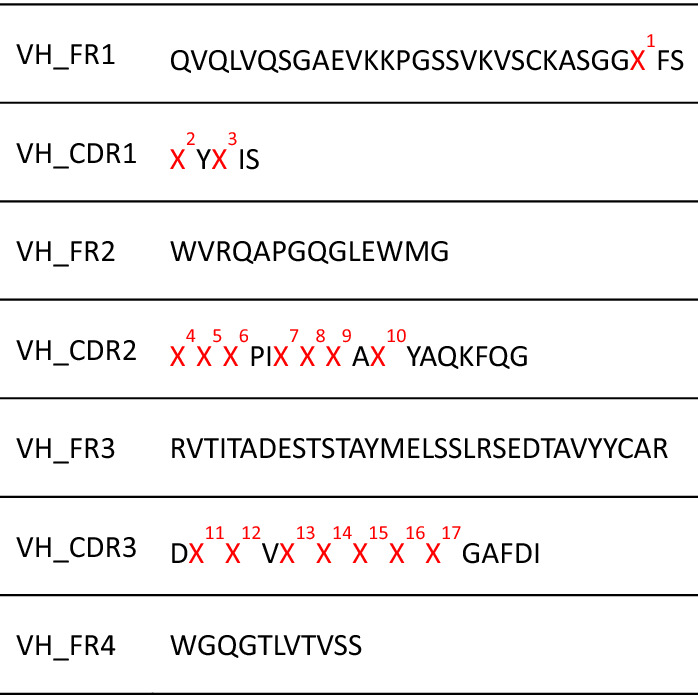
Amino acid design of F02 heavy chain library. X indicates diversified position. X^5^: I/L/V, X^8^: G/S, X^12^ and X^16^: A/P, X^17^: H/R, Other positions: 20 amino acids.

More precisely, we decided diversified positions from structural insights firstly. Surface exposed regions are diversified candidates because of relation to antigen binding. Residues responsible for forming hydrophobic core or hydrogen bond were excluded or restricted to some types of amino acids (S35, I51, I53, and R100c). Secondly, positions with few amino acids in human naïve VH repertoire were restricted (G55). We also hypothesize that proline residues have a particular relationship between other amino acids. Therefore, P97 and P100b were allowed to change into only alanine residues.

Finally, we constructed F02 alanine scanning CDR variants and determined the affinity against kynurenine by SPR. Y32, D95, V98, G100d, A100e and F100f. were excluded in our library design because their variants showed remarkable decrease of kynurenine binding.

We used five types of degenerated codon, VTT for I51, RGT for G55, SCG for P97 and P100b, CRT for R100c and NNK for other diversified positions. Theoretically, the library is composed of 2 × 10^17^ variants. We obtained more than 4 × 10^10^ transformants from this F02 phage display library.

To identify critical antibody residues for kynurenine binding and obtain training data for machine learning, we conducted two round panning against magnetic beads conjugated with biotinylated kynurenine using the phage display library. Ninety-six Fab-displayed phages picked randomly from each panning sample were prepared and the binding activities were evaluated by phage ELISA assay using kynurenine immobilized microtiter plate (Supplementary Fig. 1). The data revealed no kynurenine binding clones, defined as absorbance values were above 0.2, in the primary library. On the other hand, there were 3/96 (one round) and 23/96 (two round) anti-kynurenine clones in each panning output sample. This suggests that the sequence composition of frequent clones was oriented to kynurenine binding through the phage display panning.

**Figure 2:**
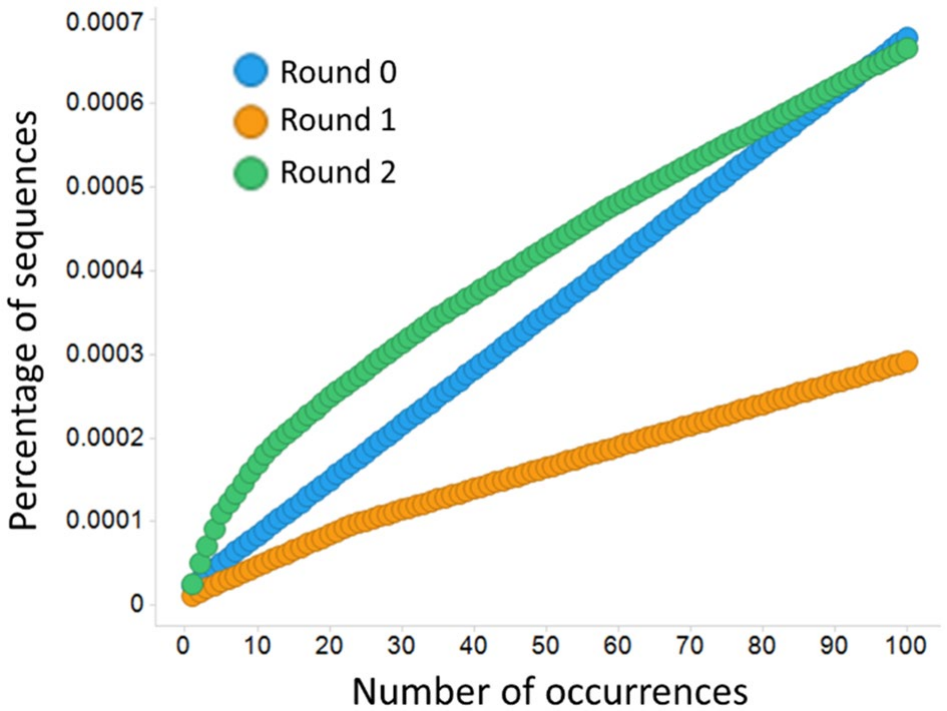
Sequence diversity of F02 library and the panning sample. Top one hundred frequent sequences from each panning sample are plotted in descending order of read counts. Vertical axis indicates accumulated percentages of NGS reads. Strong biased antibody repertoire tend to form clear curved lines.

The NGS data for the VH repertoire from panning output samples were obtained using the Miseq system, and is summarized in Table 2. We obtained more than 10^5^ in-frame and unique antibody sequences from every kind of panning rounds. The highest frequency sequences from the primary library had rates of less than 2.4 × 10^–5^ (Fig. 2, number of occurrences = 1). After panning, the values were less than 1.1 × 10^–5^ (one round) and 2.6 × 10^–5^ (two rounds). Moreover unique sequences accounted for most of the total population as percentages of unique sequences are above 0.97. This result indicates that there is enough diversity for further analysis of amino acid preferences and for machine learning.

**Table 2:** Summary of NGS data. VH genes of panning samples were analyzed by Miseq. Raw sequence reads were obtained (NGS reads). Correct sequences with antibody structure were extracted using BLAST based software (Total sequences).

Panning round	NGS reads	Total sequences	Unique sequences	Unique/Total
0	890801	453896	444170	0.979
1	912660	789338	786040	0.996
2	1227934	1083376	1051847	0.971

**Figure 3:**
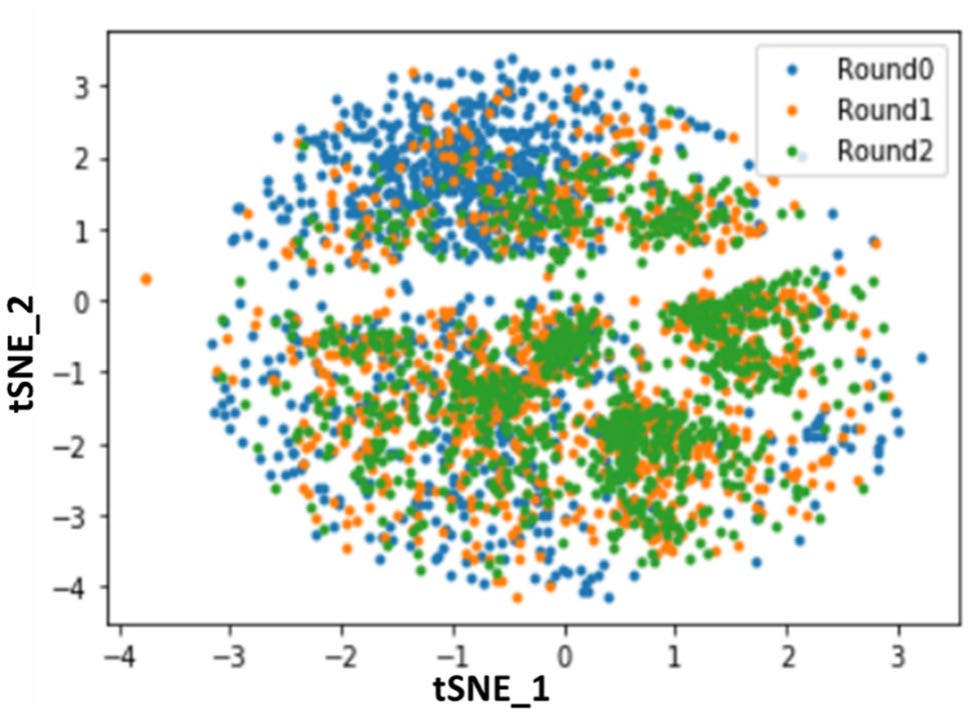
Visualization of doc2vec embedding of VH sequences by t-SNE (k-mer: 3, window size: 4, dimension: 256). Each sequence was plotted with blue (before panning), orange (one round) and green (two round).

To visualize the diversity of sequences after each panning round, we applied doc2vec to the top 1000 most frequent sequences at each round and performed dimension reduction using t-SNE^12,13^ (Fig. 3). Doc2vec was pre-trained with UniProt database as in a previous study^12^. As shown in Fig. 3, plots from single and double panning rounds were closely distributed. On the other hand, the primary library spread was more widely distributed than the single and double panning rounds. The data followed correlation between sequence feature and panning enrichment.

**Figure 4:**
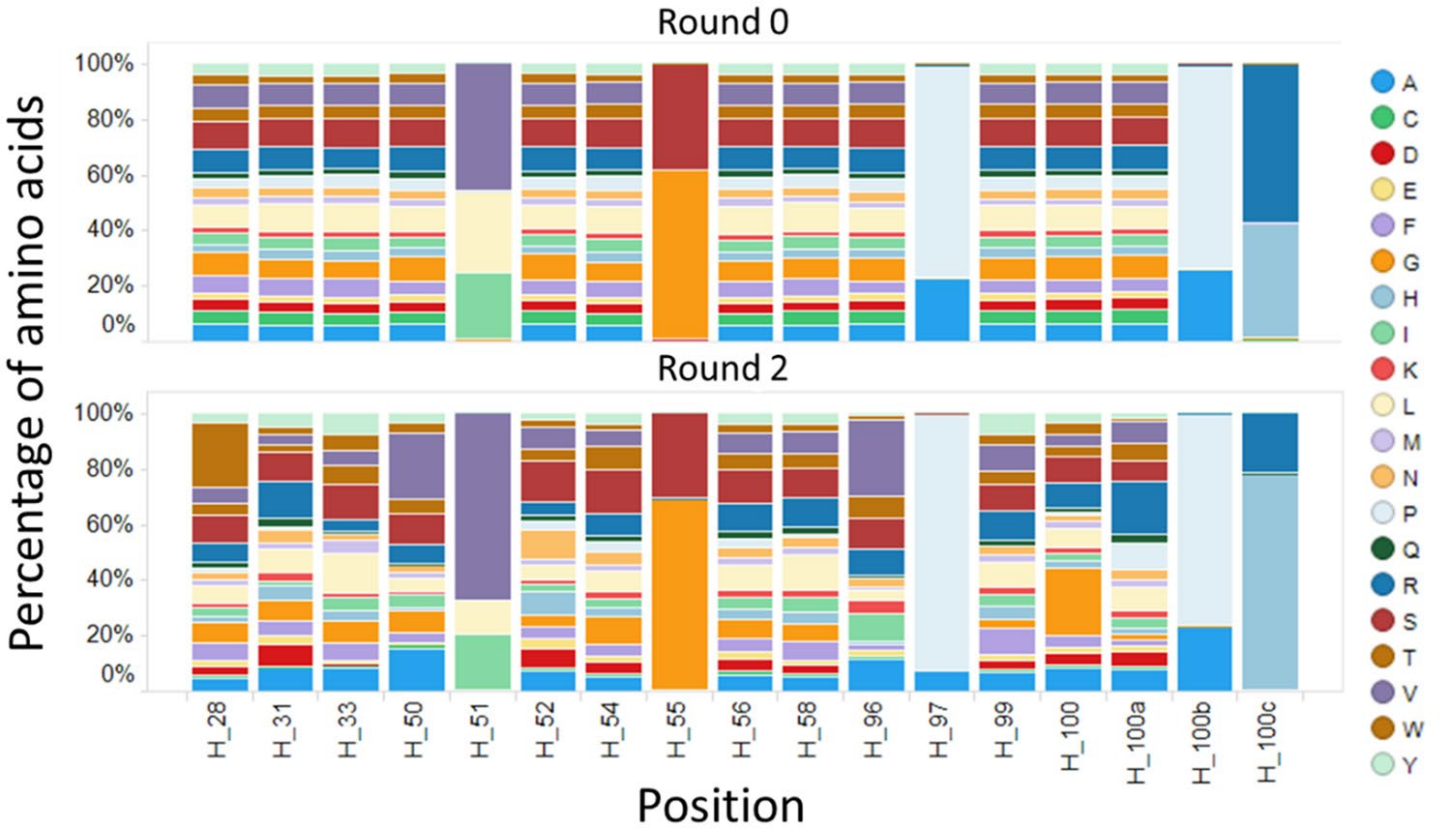
Amino acid distribution of the F02 library. The cumulative bar chart shows each composition of amino acids in diversified positions. Upper figure: primary library, lower figure: library after two round kynurenine panning.

**Figure 5:**
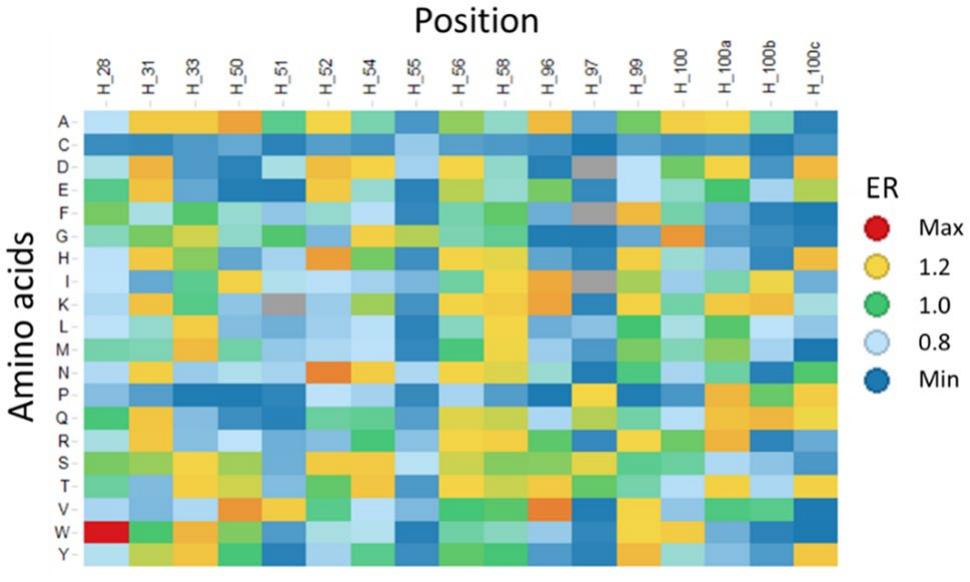
Enrichment ratio of diversified residues through panning. Enrichment ratio was calculated by NGS read counts with respect to each antibody numbering position. The ratio of amino acid compositions in diversified positions between two round kynurenine panning library and primary library is illustrated by heat-map.

We calculated the amino acid distribution for library positions (Fig. 4). The distribution of the primary library was calculated by theoretical library design. As panning proceeded, some of amino acid residues were enriched. The heat-map of enrichment ratio (ER) between the primary library and two round panning of diversified residues is illustrated in Fig. 5. ER values of 71/340 residues were over 120% and the ratio of the highest residue was 644% (T28W). ER values of 116/340 residues were neutral (0.8 < ER < 1.2) and 153/340 residues were intolerant (ER < 0.8). The data indicated that diversified residues can be characterized through phage display panning so that preferable sequences for kynurenine binding can be extracted from machine learning analysis.

### Characterizing enriched sequences and defining training sequence

**Figure 6:**
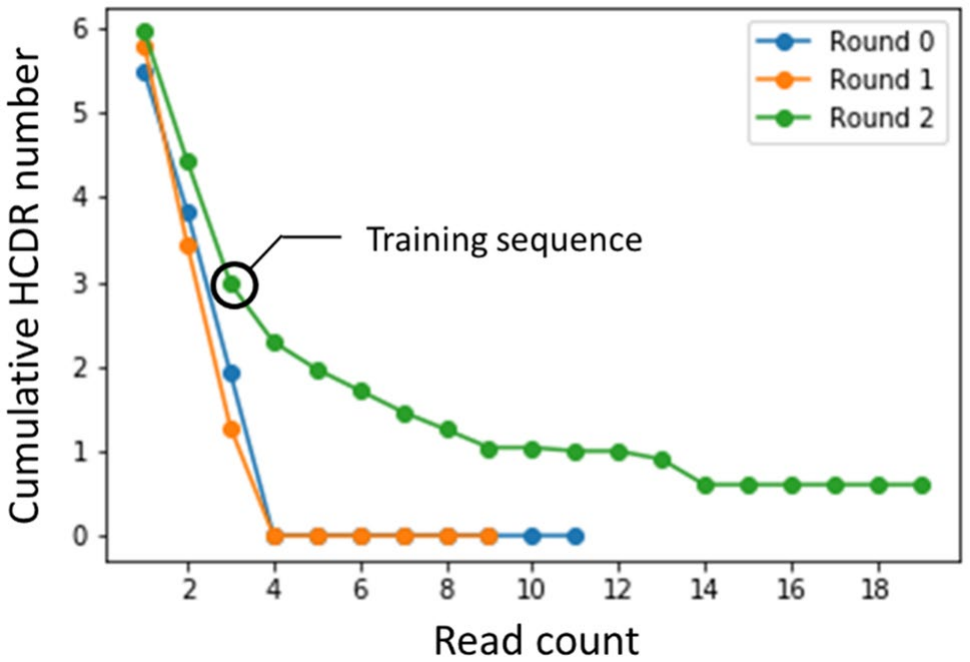
Cumulative HCDR number against read counts. X-axis and y-axis represent read counts and cumulative HCDR number, respectively. (blue: before panning, orange: one round, green: two round) are shown. Black circle indicates training sequences used in the deep generative model.

Next, we defined training sequences based on read counts from two round panning data. As shown in Fig. 6, cummulative HCDR number drastically increases for sequences where read counts are less than three. The number of sequences with more than two read counts is over 10,000, and it takes about one week to optimize the hyperparameters of a LSTM model through cross-validation. To reduce the training time of LSTM model, we selected sequences with read counts in descending order so that the number of sequences would not exceed 1000. We used the diversified residues of 959 VH sequences with over three read counts to train the model.

### Determining a LSTM model architecture and generated sequences

**Figure 7:**
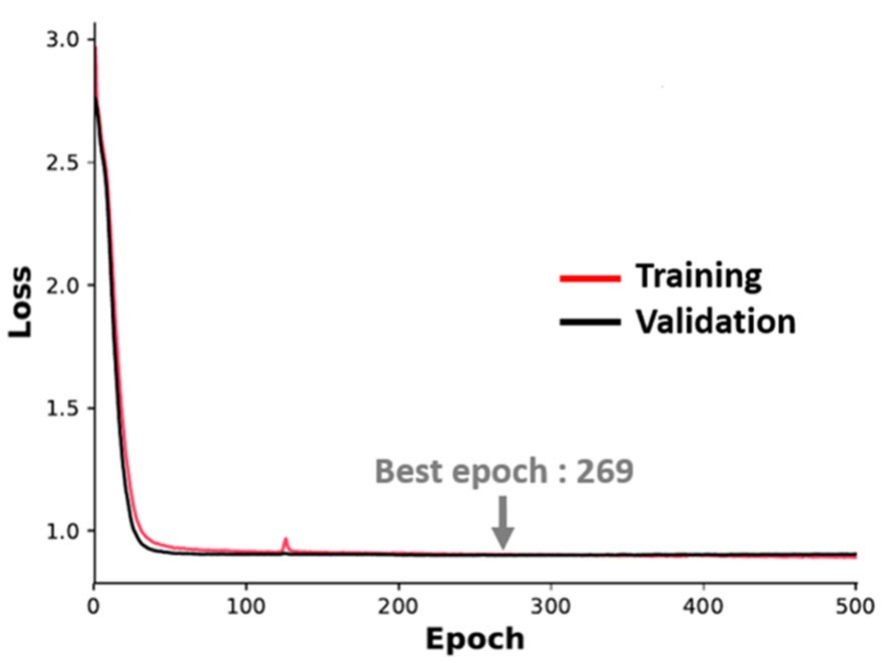
Loss evaluation of training and validation dataset. The best epoch is 269. Loss value is 0.9001. Training; red, Validation; black.

Based on the results of five-fold cross validation, we selected a network architecture with two layers containing 64 neurons and a 0.2 dropout rate. The learning curve for five-fold cross validation is shown in Fig. 7. The best validation loss was achieved at 269 epoch. Figure 7 shows that 500 epoch was enough to monitor a learning curve, because both training and validation loss converged. We used this model to generate two million new sequences.

**Figure 8:**
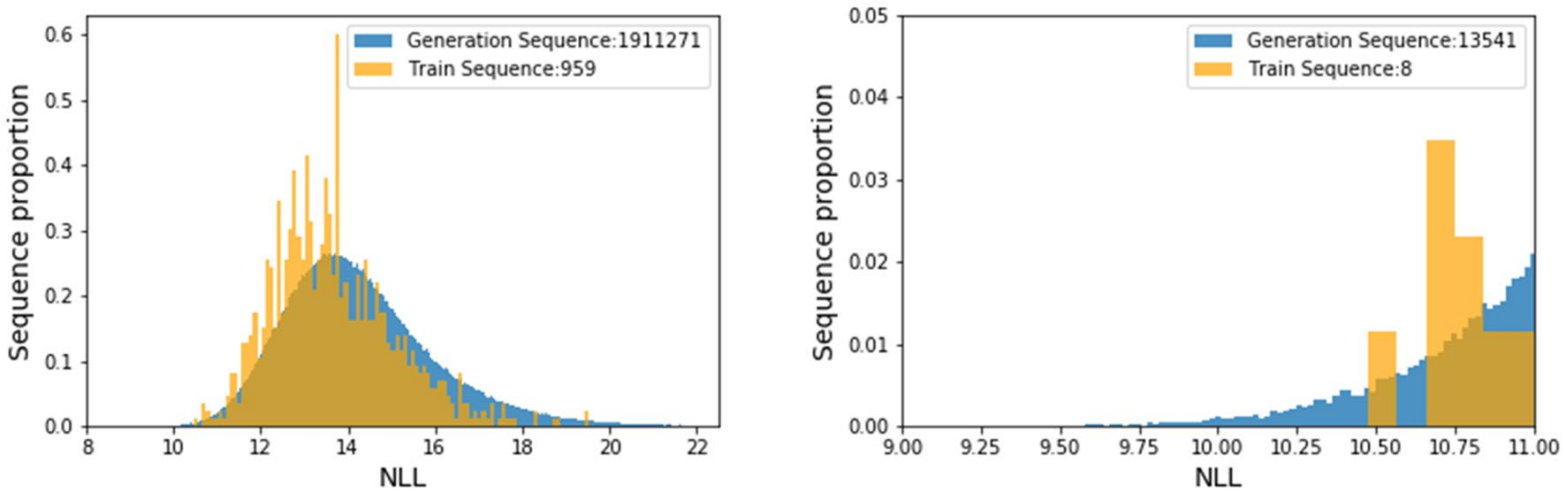
NLL distribution of training and generated sequences. NLL was calculated by applying the trained LSTM model. Enlarged view from 9.00 to 11.0 for x –axis and 0 to 0.05 for y-axis (right).

To characterize generated sequences, we calculated the NLL (see Methods for details) of generated sequences and training sequences as a prediction score (Fig. 8). The NLL histogram of generated sequences was similar to that of training sequences. This indicates that generated sequences successfully expanded the training sequence space. Moreover, the NLL of some generated sequences was lower than even the lowest training sequence. This means that there are potentially sequences with higher affinity than training sequences.

### Correlation between binding profiles and NLL

**Figure 9:**
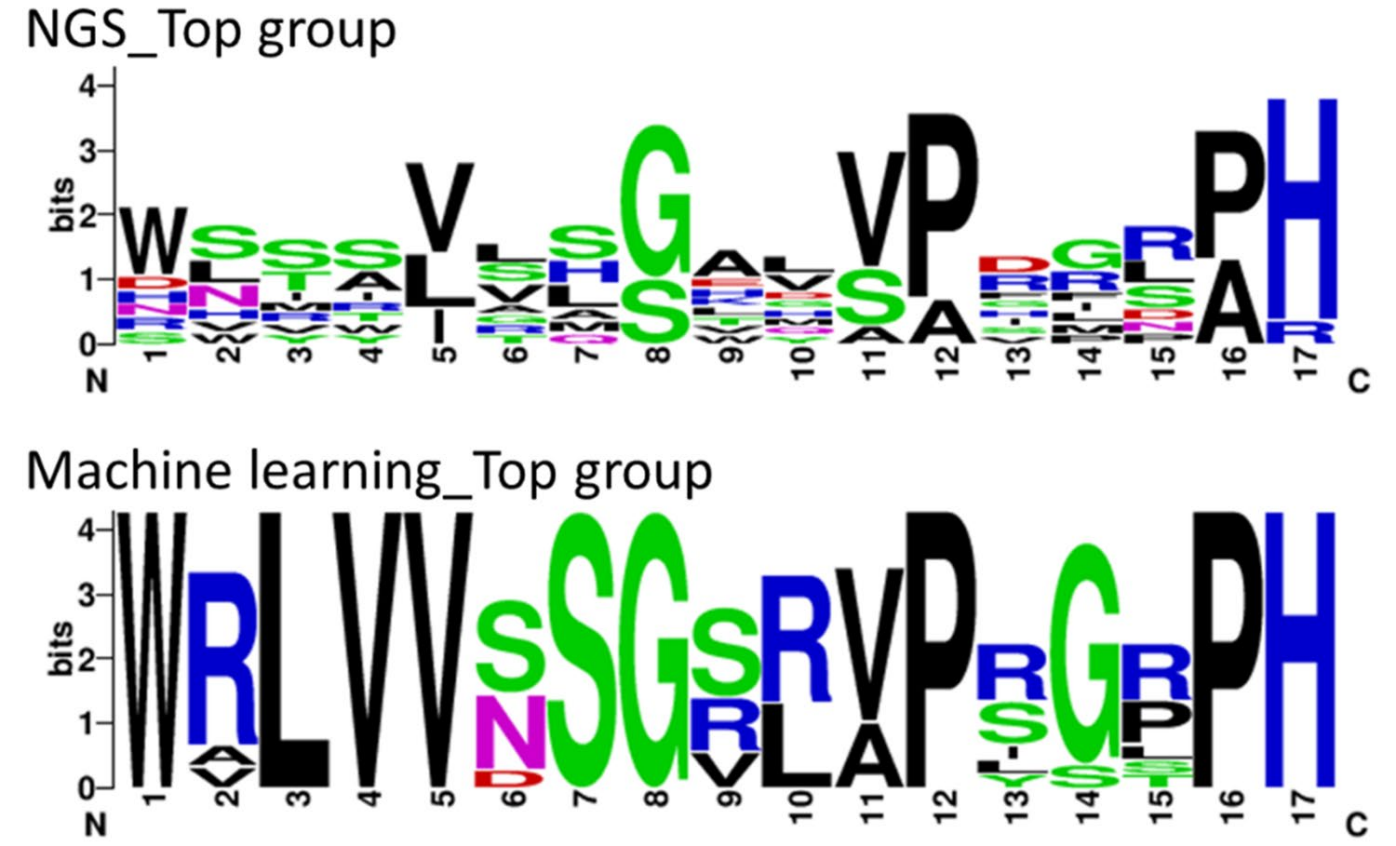
Amino acid compositions of evaluated sequences. Each diversified position is indicated in x-axis (numbering is followed by Table 1). The figures are illustrated by WebLogo (version 2.8.2, https://weblogo.berkeley.edu/).

To demonstrate that the binding affinities of generated sequences are higher than the F02 parental sequence, dissociation constants were determined by surface plasmon resonance (SPR). We selected ten sequences with the highest NLL using machine learning along with other sequences with varied NLL values to analyze the correlation between actual binding profiles and NLL (Table 3 and Fig. 9). For comparison, we also selected the ten sequences with the highest read counts from NGS data on the two round panning sample. Unique amino acid compositions were revealed in the generated sequence (e.g. X^2^: R, X^3^: L, X^4^: V, X^10^: R). All proposed sequences had expression high enough for further binding experiments (data not shown).

**Table 3:** Evaluated sequences of anti-kynurenine antibodies. The sequences are categorized into sequences derived from machine learning (ML), sequences derived from NGS total reads (Freq), and the parental sequence (Control). Residues in diversified positions are shown. Likelihood was calculated by the constructed machine learning model. Dissociation constant (K_*D*_) against kynurenine was measured by SPR.

Analysis	Position																	NLL	K_D_(M)
	28	31	33	50	51	52	54	55	56	58	96	97	99	100	100a	100b	100c		
ML	W	R	L	V	V	S	S	G	R	R	A	P	R	G	R	P	H	9.01	5.7E-07
ML	W	R	L	V	V	S	S	G	V	R	V	P	S	G	P	P	H	9.03	1.9E-06
ML	W	R	L	V	V	S	S	G	R	R	V	P	Y	G	R	P	H	9.04	9.6E-08
ML	W	A	L	V	V	S	S	G	S	L	V	P	R	G	P	P	H	9.06	9.9E-08
ML	W	R	L	V	V	N	S	G	R	R	V	P	L	G	R	P	H	9.09	5.6E-08
ML	W	R	L	V	V	N	S	G	S	R	A	P	R	G	L	P	H	9.23	7.2E-07
ML	W	R	L	V	V	N	S	G	S	L	V	P	R	G	S	P	H	9.24	6.6E-08
ML	W	R	L	V	V	D	S	G	V	R	V	P	S	G	R	P	H	9.27	4.2E-08
ML	W	R	L	V	V	S	S	G	S	L	A	P	I	G	T	P	H	9.30	4.6E-07
ML	W	V	L	V	V	N	S	G	S	L	V	P	S	S	P	P	H	9.30	8.7E-08
Freq	S	V	R	T	V	L	L	G	H	D	S	P	H	I	R	P	H	28.00	4.3E-06
Freq	W	N	V	A	I	S	L	S	A	H	S	A	V	M	D	A	H	23.00	3.9E-06
Freq	D	L	T	R	V	T	Q	G	E	V	V	P	R	L	R	P	H	22.00	3.7E-07
Freq	W	L	S	S	V	A	H	S	T	V	A	P	R	P	P	A	R	20.00	4.4E-06
Freq	W	N	T	Y	L	V	S	G	K	M	V	P	I	R	L	P	H	13.00	3.0E-06
Freq	R	W	S	I	V	V	S	G	W	L	V	P	G	G	N	P	H	13.00	9.2E-07
Freq	W	S	S	W	L	G	M	S	A	L	V	A	D	R	S	P	H	13.00	1.9E-06
Freq	N	S	I	A	I	S	A	G	L	Y	V	P	S	G	S	A	H	13.00	2.8E-07
Freq	W	S	Y	S	L	L	H	G	V	G	V	P	D	G	L	P	H	12.00	5.9E-06
Freq	H	H	M	S	V	R	S	G	A	Q	S	P	F	F	R	A	H	12.00	1.5E-05
Control	T	S	A	G	I	I	F	G	T	N	A	P	V	A	R	P	R	-	7.7E-05

**Figure 10:**
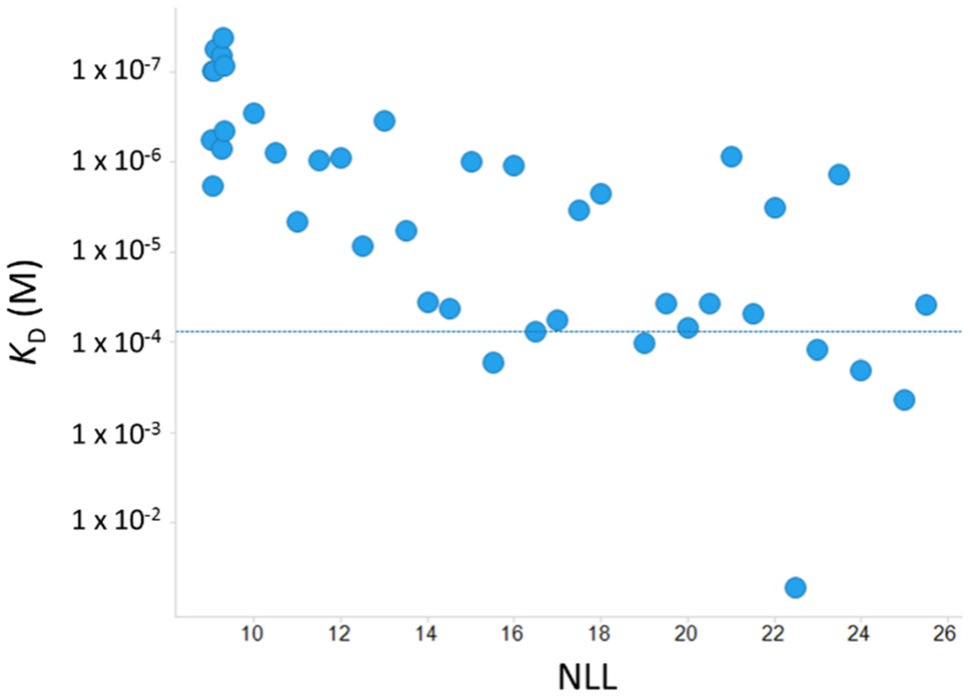
Correlation between likelihood and dissociation constant of generated antibodies from machine learning. Kynurenine binding was measured using SPR. Antibodies were captured on a recombinant protein A/G immobilized sensor chip. Kynurenine was injected as an analyte, followed by the dissociation step. Horizontal line shows the affinity of the F02 parental sequence. NLL was used as an indicator of likelihood.

**Figure 11:**
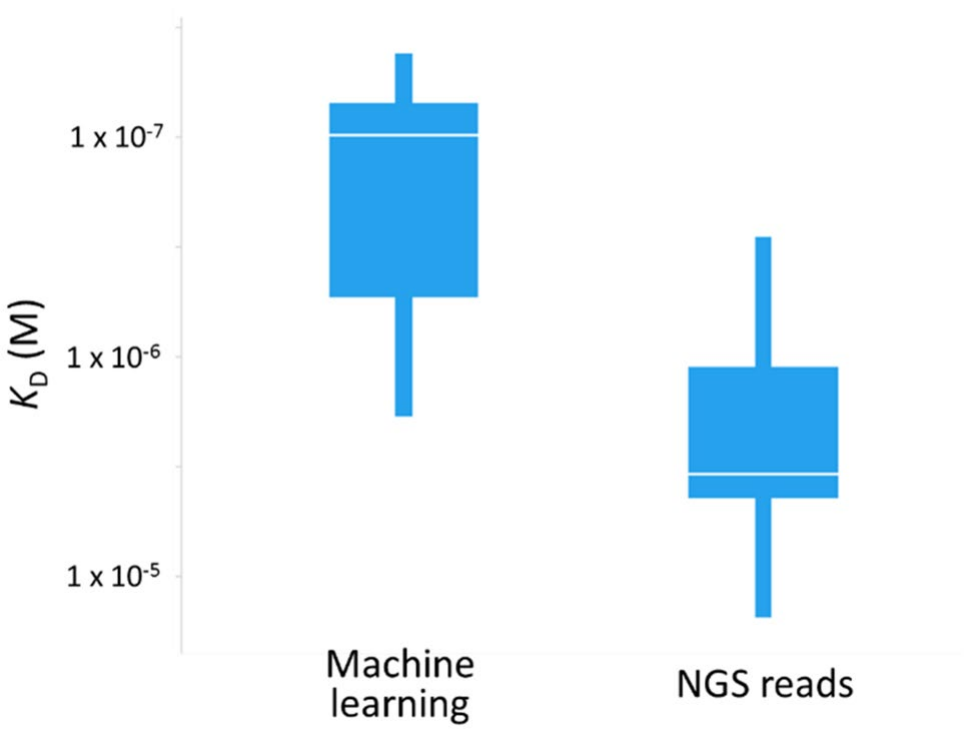
Comparison of affinity between top ten sequences derived from machine learning and NGS total reads. The data represents lower (Q1) and upper (Q3) quartiles and median (white line).

The dissociation constants against kynurenine and NLL of sequences generated through machine learning are plotted (Fig. 10). The data revealed a positive correlation between binding activity and likelihood (R^2^:0.52). The ratio of sequences with higher binding activity than the parent are 100% (20/20) (NLL < 15), 70% (7/10) (15≦ NLL < 20) and 58% (7/12) (20 ≦ NLL). A box plot of the top ten sequences identified by machine learning (NLL) and NGS reads is illustrated in Fig. 11. Median values of dissociation constants are 9.7 × 10^–8^ M (machine learning) and 3.5 × 10^–6^ M (NGS reads). The highest dissociation constants for each sequence are 4.2 × 10^–8^ M (machine learning) and 2.8 × 10^–7^ M (NGS reads). The highest affinity acquired through machine learning is more than 1800-fold higher than that of the parental clone (Supplementary Fig. 2). This result indicates that machine learning approach generates compelling results that experimental work would take longer and not as efficiently to achieve.

## Discussion

In summary, we have developed a machine learning platform for antibody affinity maturation. We applied it to anti-hapten antibody screening and were able to generate antibody sequences with well-correlated NLL and binding affinity. Their affinity was also improved over 100-fold, surpassing what is possible with frequency based screening using the same dataset.

We recently developed a novel antibody technology referred to as “Switch-Ig.” An antibody using this technology only binds to a target antigen when specific metabolites are highly accumulated. This “switch” feature is utilized in disease-related microenvironments, such as tumor tissues, to enlarge the therapeutic window. For example, we engineered ATP dependent anti-CD137 antibody for cancer immunotherapy^14^. For development of this type of antibodies, we should conduct protein engineering based on anti-target metabolite antibodies (anti-hapten antibodies). As a consequence, more antibodies can be engineered to bind with small molecules, thus expanding the reach of conventional immunotherapy.

In antibody discovery, any approach targeting haptens is prone to be harder, and our method achieves a similar outcome without dependence on specific library designs or antigens. Our scheme can be also applied to antibody screening against any target, including protein antigens.

To achieve successful results, our method differs from previous approaches in several key ways. For improving affinity, our scheme can also explore virtual sequences beyond the phage display library size. There are theoretically 2 × 10^17^ unique sequences in our combinatorial library, which is more than in the actual library consisting of 4 × 10^10^ transformants. Moreover, sequencing reads are fewer than one million. With machine learning, we can extract all the valuable residues from limited NGS data simultaneously and incorporate them into generated sequences. NGS-based site saturation mutagenesis is another option for solving the problem. It is reported that high-affinity antibodies were obtained through analysis of the NGS derived enrichment ratio by the residue^5^. In that report, a double mutant showed strong antigen binding although single mutations by themselves had a negative impact^15,16^. Machine learning might reveal not only additive but also synergistic mutation pairs for antigen binding. Another advantage of machine learning is that the sequence, which has low expression in the phage display, can be evaluated virtually.

In terms of sequence space, it is generally difficult to enumerate and evaluate comprehensive CDR sequences because of combinatorial explosion. Therefore, previous deep learning approaches were restricted to limited sequence space (e.g. HCDR3). In contrast, we achieved efficient sequence sampling using a deep generative model, LSTM. Moreover, with the LTSM model, likelihood-based scoring is not needed to distinguish between binders and non-binders for training data. Moreover, with NGS data analysis and the deep learning model, the progress of sequence enrichment is monitored through phage display panning, which we therefore used to select appropriate training samples for precise prediction.

There are several related examples of how the machine learning technique has been applied to antibody sequencing. Liu applied a convolutional neural network (CNN)-based regression model to enrich panning, and used a gradient-based technique to optimize sequences^6^. They reported that machine learning-designed sequences had higher affinity than panning-derived sequences. In contrast, our method incorporates only enriched sequence information and does not require panning as a control group, making it quite different from Liu’s method. In antibody optimization, Mason used CNN and LSTM to train a classification model that discriminates binders and non-binders for a CRISPR/Cas9 mediated homology-directed mutagenesis repair system^7^. They aimed to optimize multiple kinds of developability parameters such as affinity, viscosity, solubility and immunogenicity for discovering highly-optimized lead candidates. In contrast, our study focuses on likelihood-based prioritization and efficient sequence generation. Therefore, the direction of the study is quite different. In terms of likelihood-based prioritization, DeepSequence is conceptually similar to ours^17^. DeepSequence aimed to find thermostable mutants from multiple sequence alignment by evidence lower bound (ELBO) for variational autoencoder (VAE). This ELBO for VAE is similar to our NLL for LSTM, because both indicators calculate sequence likelihood using a trained model; however, the aim of our study is completely different.

In the future, there is a room for improvement in the tuning of each step. For example, we should select true binder sequences from NGS data for the training data set. For obtaining anti-bevacizumab specific antibodies, Liu conducted panning against not only bevacizumab, but other fc-containing antibodies as well. Antibody sequences with target specific enrichment were used for training data. Moreover, They also constructed a non-specific binding model using panning samples against fc-containing antibodies other than bevacizumab to reduce the number of non-specific binder sequences^6^. Another possible indicator of true binder sequences is enrichment ratio between panning rounds^18^. Although we have not tried to optimize panning condition because our method is robust, we can also modify panning antigen concentration and washing round for precise analysis. Based on our current knowledge, the development of universal definition flow is preferable for precise analysis.

To apply our method to other unmet needs, we will need to develop peripheral technologies for data acquisition and analysis. Although this particular study only used a synthetic heavy chain library with limited sequence diversity, the method could also be applied to antibody discovery using a universal antibody library (e.g., human naïve or synthetic library) and immunized antibody repertoire (e.g., mouse, rabbit). First, in terms of data acquisition, precise paring of heavy and light chains is important for predicting antigen binding^19^. There are various experimental and analytical approaches that can be used to retain this precision, such as frequency based ranking^20^, utilization of a long read sequencer^21^, single cell analysis by droplets^22^, and CDR recombination^23^. Second, antibody structure and the role of residues in variable regions have been clarified based on some common numbering schemes from the analytical point of view^24^. The alignment of amino acids in CDRs might be critical for precise encoding using an appropriate algorithm^25^. Although we used panning derived NGS data, sequences have different paratopes and epitopes against target antigens. Classification of antibody repertoire is important for predicting structural similarity^26^ and biological activity^27,28^.

In this study, high quality sequences were generated based on the probability of true binders. When specific features extracted from NGS data are correlated with quantitative binding affinity, we are also able to predict the affinity of sequences. Moreover, some reports describe schemes for screening other characteristics, such as thermal stability^29^. Thus, our approach can be broadly applied to a variety of issues in protein engineering.

Methods.

### Construction of F02 Hch library

DNA sequences in VH and VL regions of F02 antibody clone were incorporated into a phagemid vector with human CH1-geneIII fusion and human CL genes. Degenerated oligonucleotides were designed to contain diversified heavy chain variable regions and overlapping fragments for PCR primer binding. The oligonucleotide library was synthesized and incorporated into the phagemid vector. The phagemid library was transformed into Escherichia coli, ER2738 (Lucigen Corporation, USA), by electroporation. The transformants were cultured in 2 × YT medium, then infected with Hyperphage (Progen Biotechnik, Germany). After overnight cultivation, the phages were purified by PEG precipitation.

### Panning

Biotinylated kynurenine was chemically synthesized as panning antigen. In round one, 6 nmol of antigen was immobilized on streptavidin-coated magnetic beads (Thermo Fisher Scientific, USA). The beads were blocked by TBS/4%BSA. Phage library was mixed with the antigen-coated beads in 800 μL TBS/4%BSA. The supernatant was washed out by TBS /0.1% Tween20 twice and TBS once. Bound phage were eluted by trypsin digestion and re-infected into ER2738 cells. In round two, 10 nmol of antigen was immobilized on neutravidin-coated magnetic beads (GE healthcare, USA). Reaction supernatant was washed out by TBS /0.1% Tween20 three times and TBS twice. The other process in round two was the same as in round one.

### Phage ELISA

ER2738 cells electroporated or infected by panning output phage were plated on a 2 × YT agar plates. Ninety six colonies from each panning condition were selected. The clonal cells were cultured and infected with Hyperphage. After overnight cultivation, the culture supernatant was collected. For the ELISA assay, biotinylated kynurenine was immobilized on a streptavidin-coated plate. Phage supernatant was incubated in the antigen-coated plate for 1 h. The bounded phages were reacted with HRP conjugated anti-M13 phage antibody (GE healthcare, USA) and TMB solution as substrate reagent. Absorbance of 450 nm was measured as binding signal.

### Illumina sequencing

Phagemid DNA was extracted from ER2738 cells electroporated or infected by panning output phage with QIAprep Spin Miniprep Kit (Qiagen, Germany). Genes encoding the heavy chain variable region were amplified with barcodes by PCR and purified by Labchip system (PerkinElmer, USA). The samples were sequenced on an Illumina Miseq system according to manufacturer’s protocol.

### Fastq data processing

NGS reads were reconfigured by detecting a match between each forward and reverse read for paired end assembly. Assembled reads are classified into each sample group by barcode sequence. DNA sequences were translated into amino acid sequences with three forward frames. Afterwards, VH sequences were identified using BLAST based methods.

### LSTM model architecture and training procedure

**Figure 12:**
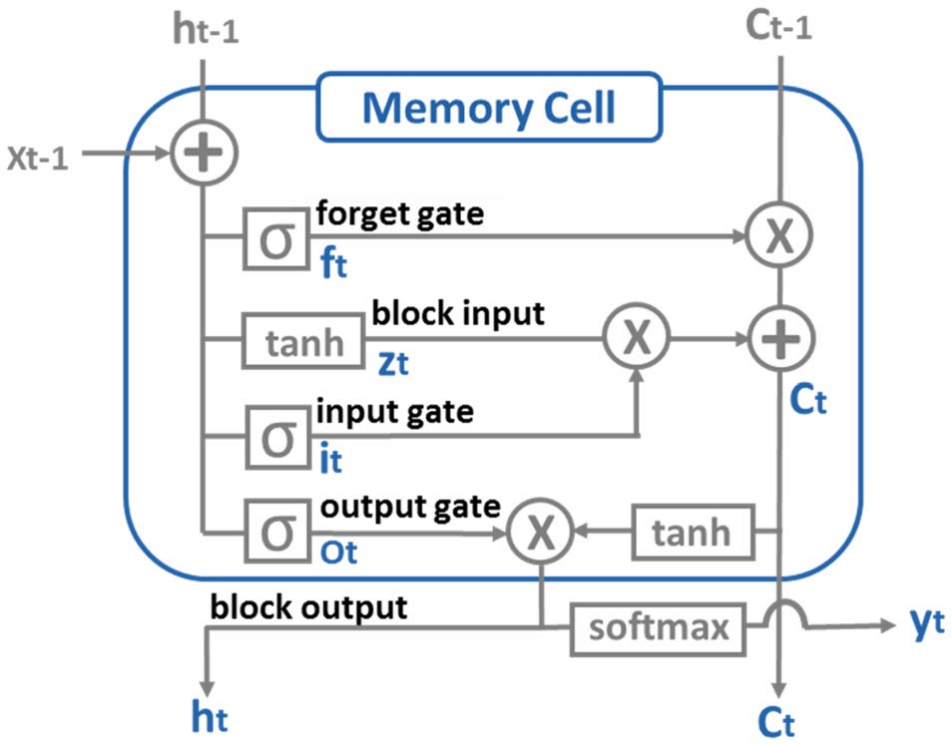
Illustration of LSTM architecture. LSTM is consisted of block input, input gate, forget gate, output gate, block output, and memory cell. Computed one hot vector is outputted through the softmax function.

An LSTM model is a class of recurrent neural network (RNN), which is the one of the most popular tools in the natural language processing and speech recognition field^30,31^. RNN can capture sequence information and can handle sequences of arbitrary length. However, the original RNN suffers from the problem of vanishing or exploding gradients in backpropagation training. To address this, LSTM architecture is composed of three gates (input, forget, output), block input and a memory cell (the constant error carousel) that allows the network to learn when to forget the previous hidden states and when to update hidden states based on new input. To our best knowledge, our study is the first case applying LSTM to antibody sequences. The LSTM architecture is illustrated in Fig. 12. Let $x_{t} \in \left\{ {0,1} \right\}^{A}$ be the input one-hot encoding vector at position t, where A is the number of vocabulary, i.e., 22, that consists of 20 natural amino acid letters, a start token, and a padding token. Suppose that N is the number of LSTM blocks. We define the following weights for an LSTM layer:

- Input weights: $${\text{W}},W_{i} ,W_{f} ,W_{o} \in R^{N \times A}$$
- Recurrent weights: $$R_{z} ,R_{i} ,R_{f} ,R_{o} \in R^{N \times N}$$
- Bias weights: $$b_{z} ,b_{i} ,b_{f} ,b_{o} \in R^{N}$$

Then, the vector formula for a LSTM layer forward pass can be written as the following:

- Block input: $$z_{t} = tanh\left( {W_{z} x_{t} + R_{z} h_{t - 1} + b_{z} } \right)$$
- Input gate: $$i_{t} = \sigma \left( {W_{i} x_{t} + R_{i} h_{t - 1} + b_{i} } \right)$$
- Forget gate: $$f_{t} = \sigma \left( {W_{f} x_{t} + R_{f} h_{t - 1} + b_{f} } \right)$$
- Memory cell: $$c_{t} = z_{t} *i_{t} + c_{t - 1} *f_{t}$$
- Output gate: $$o_{t} = \sigma \left( {W_{o} x_{t} + R_{o} h_{t - 1} + b_{o} } \right)$$
- Block output: $$h_{t} = o_{t} *{\text{tanh}}\left( {c_{t} } \right)$$

The symbols $h_{t - 1}$ and $h_{t}$ represent the outputs of the previous memory cell and the current one, respectively. ${\upsigma }$ is a sigmoid function (${\upsigma }\left( x \right) = 1/1 + e^{ - x}$) and is used as gate function. The hyperbolic tangent function (tanh) is used as the block input and output activation function. Hadamard product of two vectors is represented by ${*}$. LSTM adaptively passes information through a gate unit by a sigmoid layer and a pointwise multiplication operation. Sigmoid layer output ranges from 0 to 1, and it represents the weight that the corresponding information passes through. In other words, 0 means no information is allowed, and 1 means all information is passed.

The output of the LSTM layer is connected with a densely connected feed-forward layer combining the output signals with a softmax function. Softmax function is introduced to restrict the summation of the output to 1. We employ the categorical cross-entropy loss function L between the predicted and the actual target vectors to calculate for every one-hot encoded residue in an amino acid sequence with K length.

$$ {\text{L}}\left( {t,y} \right) = - \mathop \sum \limits_{k = 1}^{K} t_{k} log\left( {y_{k} } \right) $$

where $y_{k}$ is the predicted *k*-th one-hot vector from softmax layer and $t_{k}$ is the true target *k*-th amino acid vector in the training data. To minimize the loss function, we used the Adam optimization algorithm with a learning rate of 0.01^32^. To determine appropriate hyperparameters for the model, we performed five-fold cross validation with different LSTM architectures over 500 epoch. The number of LSTM blocks was chosen from [24, 32, 64, 128, 256, 512] for one or two LSTM layers. Dropout rates were chosen from 0.1 and 0.2 to regularize all layers. For all architectures, we determined the epoch at which validation loss was minimized. The validation loss at this epoch is used as the criterion for selecting the best LSTM architecture. We implemented the LSTM model in Python using Keras^33^ (version 2.0.2) with the Tensorflow^34^ (version 1.3.0) backend.

### Likelihood for estimating binding affinity

To train a LSTM model that can generate sequences which tend to be antigen binders, we first trained LSTM using binder sequences, where the binder sequences were defined as those whose NGS occurrence was higher in round 2 than in 3. This model learned the characteristics of binder sequences, and is expected to generate sequences which tend to bind antigens.

We also assumed that likelihood, which can be calculated from the trained model, would correlate with binding affinity. We proposed a negative logarithm of likelihood (NLL) for a sequence based on a learned LSTM model using the following formula:

$$ {\text{NLL}} = - \mathop \sum \limits_{k = 1}^{K} log\left( {p\left( {x_{k}^{{}} } \right)} \right) $$

where $p\left( {x_{k}^{{}} } \right)$ represents the generative probability of a letter at k-th position. When $p\left( {x_{k}^{{}} } \right)$ is large, a letter of k-th position frequently appears in the training sequences. Consequently, NLL becomes small when a lot of $p\left( {x_{k}^{{}} } \right)$ is near to 1. We assume that the smaller the NLL, the stronger a sequence binds to an antigen. We show that this assumption is valid using real panning NGS data.

### Sequence generation

We determine the best LSTM model through five-fold cross validation for sampling new sequences. When generating new sequences, we begin with the start token, and then we continue to sample amino acid characters until we reach the maximal sequence length. To control generated sequence diversity, we introduce a temperature factor into the softmax function. The generative probability with temperature factor $P_{k}^{i}$ for selecting *i*-th amino acid at position *k* is defined as the following.

$$ P_{k}^{i} = \frac{{exp\left( {y_{k}^{i} } \right)/T}}{{\mathop \sum \nolimits_{{i^{\prime} = 1}}^{A} exp\left( {y_{k}^{{i^{\prime}}} } \right)/T}} $$

If we set T to over 1, we can sample more diverse sequences. On the other hand, if we set T under 1, we only sample biased sequences. We consecutively generate sequences according to the above generative probability. In this study, we set T to 1, and sampled 2 million sequences. After generating sequences, we removed those that had an amino acid in positions not seen in training sequences.

Antibody sequences for experimental evaluation were proposed using the trained model in the following three groups: (1) 10 lowest NLL sequences generated from LSTM model, (2) 32 sequences with NLL in the range of 10 to 25.5 in 0.5 steps, (3) 10 most frequent sequences based on NGS reads from two round panning.

### Surface plasmon resonance (SPR)

Generated antibody sequences from NGS data and machine learning were synthesized and incorporated into mammalian expression vector. Recombinant antibodies were expressed transiently in FreeStyle™ 293F cells (Invitrogen, USA) and purified from cultured medium using protein A. The antigen binding levels were measured using a Biacore 8 K + instrument (GE healthcare). Antibodies were captured on a recombinant protein A/G (Thermo Fisher Scientific) immobilized CM5 sensor chip. Kynurenine diluted in running buffer (20 mM ACES, 150 mM NaCl, pH 7.4, 0.05w/v% Tween20) was injected, followed by the dissociation step. The response signal was obtained by subtracting the antibody uncaptured flow cell response from the antibody captured flow cell response, and the difference of each response signal with and without kynurenine solution was calculated as normalized response. Kinetic analysis was performed with a steady state affinity model using Biacore Insight Evaluation Software (GE healthcare).

## Supplementary Information

Supplementary Information.

Supplementary Fig. 1.

Supplementary Fig. 2.
