# Supplementary material for: Antibody design using LSTM based deep generative model from phage display library for affinity maturation
Source: Sci Rep. 2021 Mar 12;11:5852. doi: 10.1038/s41598-021-85274-7 (PMC7955064; doi:10.1038/s41598-021-85274-7)
Supplement: Supplementary file 1 — Supplementary Information. [file 41598_2021_85274_MOESM1_ESM.docx]

Title

Antibody design using LSTM based deep generative model from phage display library for affinity maturation

Authors

Koichiro Saka^1^, Taro Kakuzaki^1^, Shoichi Metsugi^1^, Daiki Kashiwagi^2^, Kenji Yoshida^1^, Manabu Wada^1^, Hiroyuki Tsunoda^1^ & Reiji Teramoto^1,*^

Affiliations

^1^Research Division, Chugai Pharmaceutical Co., Ltd., Kamakura, Kanagawa, Japan, ^2^Research Division, Chugai Pharmaceutical Co., Ltd., Gotemba, Shizuoka, Japan, Koichiro Saka and Taro Kakuzaki contributed equally to this work, ^*^email: teramoto.reiji11@chugai-pharm.co.jp,

Supplementary Figure 1

Phage ELISA against kynurenine. 96 transformants were picked up from each panning sample. Each Fab displayed phage were expressed and collected from the clonal transformants. Phages bind to kynurenine coated microtiter plate. Binding activity was detected by HRP conjugated anti-M13 antibody. Data derived from each clone were plotted. Vertical axis indicates absorbance as binding activity against kynurenine.

Supplementary Figure 2

SPR sensorgram. Binding responses of parent control and the best maturated antibody through machine learning were shown. The Response was indicated as resonance unit (RU). X-axis shows elapsed time from analyte injection. There are nine analyte concentration points (0.14~900 μM: Parent, 0.0051~33 μM: Best maturated). The figures are illustrated by Biacore Insight Evaluation Software.
