# Supplementary figures and images for: Antibody design using LSTM based deep generative model from phage display library for affinity maturation
Source: Sci Rep. 2021 Mar 12;11:5852. doi: 10.1038/s41598-021-85274-7 (PMC7955064; doi:10.1038/s41598-021-85274-7)

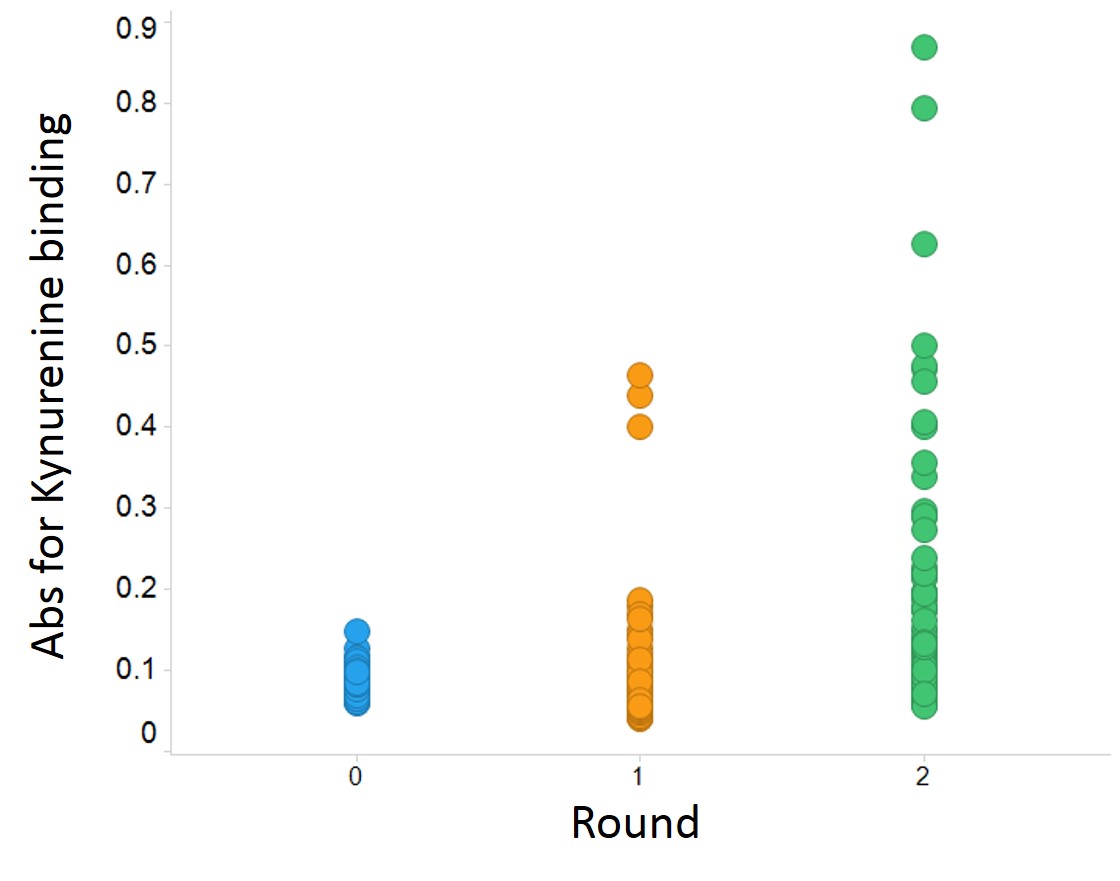

Supplement: Supplementary file 2 — Supplementary Fig. 1. [file 41598_2021_85274_MOESM2_ESM.jpg]

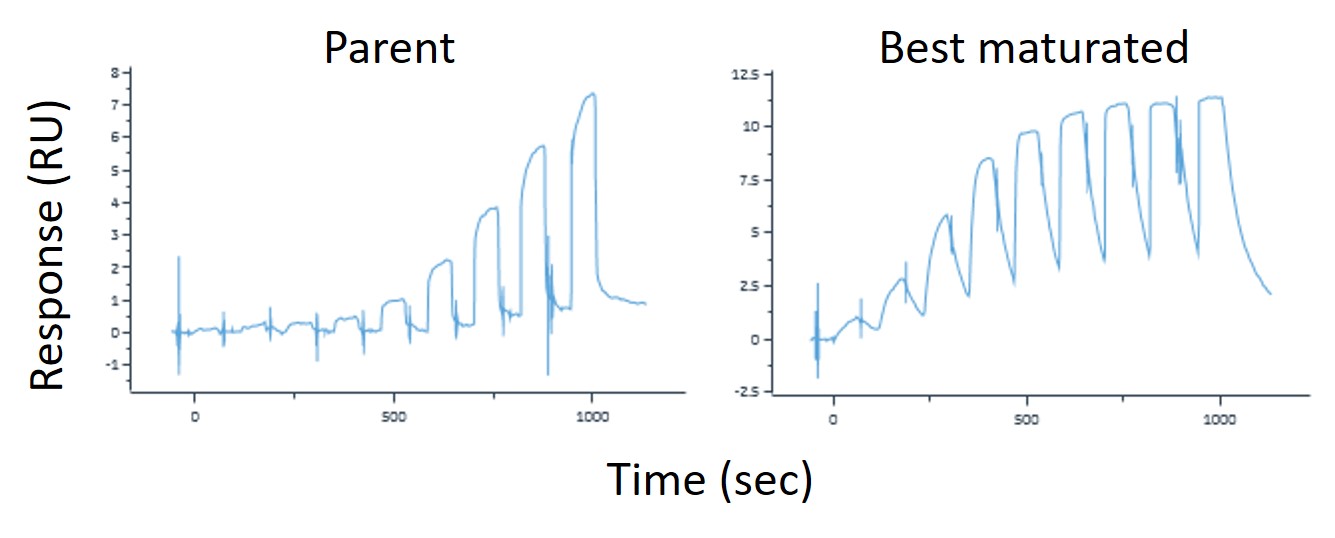

Supplement: Supplementary file 3 — Supplementary Fig. 2. [file 41598_2021_85274_MOESM3_ESM.jpg]
